# Infinitely large, randomly wired sensors cannot predict their input unless they are close to deterministic

**DOI:** 10.1371/journal.pone.0202333

**Published:** 2018-08-29

**Authors:** Sarah Marzen

**Affiliations:** Department of Physics, Physics of Living Systems Group, Massachusetts Institute of Technology, Cambridge, MA, United States of America; Arizona State University & Santa Fe Institute, UNITED STATES

## Abstract

Building predictive sensors is of paramount importance in science. Can we make a randomly wired sensor “good enough” at predicting its input simply by making it larger? We show that infinitely large, randomly wired sensors are nonspecific for their input, and therefore nonpredictive of future input, unless they are close to deterministic. Nearly deterministic, randomly wired sensors can capture ∼ 10% of the predictive information of their inputs for “typical” environments.

## Introduction

Prediction is thought to be fundamental to organism functioning, e.g. see Ref. [1] and references therein. By better predicting the world around them, organisms can better choose actions that will maximize their reaped reward. On the other hand, prediction of the future of a time series from its past drives much of science, e.g. the realization that one could predict future particle positions from some parameters (such as particle masses and charges) and current particle positions and velocities.

So how should one build predictive time series models? One common trick is to feed the data we wish to predict into a recurrent network, the state of which can contain enough memory of the past in order to be predictive of the future. Sometimes, these networks are trained so that the parameters defining the network’s dynamics yield optimal predictions of the input time series, e.g. as in Ref. [2] and references therein. However, much utility has been gained from using randomly connected networks (or reservoirs) [3–6], where one merely trains the readout of the network. Such networks can have nearly maximal predictive power if the networks are large enough [7]. Such a finding might also imply that perhaps, evolution need not work so hard to build predictive networks (or sensors) in organisms; rather, randomly wiring large biological sensors could lead to sufficiently good predictive performance.

Here, we identify a key sensor property without which the sensor has little predictive power: determinism. Determinism means that the present sensor state and the present input state uniquely determine the future sensor state. To be clear, the sensors studied in Refs. [3, 4, 6] are deterministic as long as there is no added noise, and so the effect of nondeterminism on predictive capabilities of recurrent networks/reservoirs is somewhat unstudied. Interestingly, we find that the detrimental effects of nondeterminism are compounded rather than mitigated by large sensor size when the sensor has recurrent connections.

We find numerical and analytical evidence that nondeterminism greatly limits the ability of a (recurrent) randomly wired sensor to be predictive of its inputs, due to the weak law of large numbers. For some nondeterministic, randomly wired sensors, there is a finite optimal sensor size at which predictive power is maximized. This optimal sensor size seems to balance the larger predictive capacity of larger sensors against a trend towards nonspecificity for input demanded by the weak law of large numbers.

When the sensors connections are nearly deterministic, then larger sensors are on average more predictive. Large, deterministic, randomly wired sensors can capture ∼ 10% of the total predictive information possible. This is comparable to the ∼ 20% of predictive information captured by sensors whose weights have been (locally) optimized via the BFGS algorithm.

## Setup

First, we discuss the model of the environment, which is given by the output of a unifilar Hidden Markov model. Then, we discuss the model of the sensor, specifying notation for the dynamics of a conditionally Markovian discrete-valued sensor. Finally, we describe metrics for sensor performance: memory; and predictive information captured.

Throughout what follows, we characterize time series and the relations between them via entropy and mutual information. The entropy of a random variable *X* with realizations *x* and probability distribution *Pr*(*X* = *x*) is given by

$$\begin{array}{c} H\left[X\right]=-\sum_{x}Pr\left(X=x\right)\log Pr\left(X=x\right), \end{array}$$ (1)

while the mutual or shared information between a random variable *X* and a random variable *Y* with realizations *x* and *y*, respectively, and joint probability distribution *Pr*(*X* = *x*, *Y* = *y*) is given by

$$\begin{array}{c} I\left[X;Y\right]=\sum_{x,y}Pr\left(X=x,Y=y\right)\log\frac{Pr(X=x,Y=y)}{Pr(X=x)Pr(Y=y)}. \end{array}$$ (2)

One can think of entropy as a measure of the uncertainty of a random variable. Maximally uncertain random variables have a uniform distribution over values, and this maximizes entropy; minimally uncertain random variables are singly supported, and this minimizes entropy. One can think of mutual information as a measure of the nonlinear dependency of two random variables. From the identity *I*[*X*;*Y*] = *H*[*X*] − *H*[*X*|*Y*], mutual information is the reduction in uncertainty about a random variable *X* that comes from knowing a potentially related random variable *Y*. Operational meanings of entropy and mutual information come from Shannon’s source coding and noisy channel coding theorems [8, 9].

### Model of environment

To test the predictive capabilities of sensors, we wish to construct a non-Markovian environment that is at least somewhat predictable. The non-Markovianity of the environment will force a predictive sensor to remember longer and longer pasts, while the predictability of the input will guarantee that remembering the “right” things about environmental pasts leads to measurable predictive gains. Ideally, it would also be difficult to infer the right predictive features, so that the environments provide a challenge for sensors that desire to be predictive of their input. Environments generated by random minimal unifilar Hidden Markov models (or *ϵ*Ms [10]) as specified below turn out to satisfy all these requirements.

We consider randomly generated binary-alphabet environments, that is, environments generated by a randomly-drawn unifilar Hidden Markov model. Here, $S_{t}$ is the random variable for the hidden state at time *t*, while *X*_*t*_ is the random variable for the observed symbol at time *t*. An edge-emitting discrete-time Hidden Markov model is specified by a set of internal “hidden” states *σ* ∈ **S**, a set of possible observables x∈X, and a labeled transition dynamic $Pr(S_{t+1}=\sigma^{\prime },X_{t}=x|S_{t}=\sigma )$. For the purposes of this paper, X={0,1}. When **S** is countable, we can represent the labeled transition dynamic by a set of labeled transition matrices *T*^(*x*)^ with elements

$$\begin{array}{c} T_{\sigma^{\prime },\sigma }^{\left(x\right)}:=Pr\left(S_{t+1}=\sigma^{\prime },X_{t}=x|S_{t}=\sigma \right). \end{array}$$ (3)

A unifilar Hidden Markov model is one such that $Pr(S_{t+1}|X_{t}=x,S_{t}=\sigma )$ is singly supported, i.e. one for which the current state and symbol emitted uniquely specify the next state. This is a version of determinism in that the next state is uniquely determined, but the next symbol is not uniquely determined.

To randomly generate environments, we randomly generate labeled transition matrices *T*^(*x*)^ as follows. In each hidden state *σ*, we choose $\sum_{\sigma^{\prime }}T_{\sigma^{\prime },\sigma }^{\left(0\right)}$ (the probability of emitting a 0) uniformly at random from the unit interval; this specifies, for binary-alphabet processes, $\sum_{\sigma^{\prime }}T_{\sigma^{\prime },\sigma }^{\left(1\right)}$ (the probability of emitting a 1). We then randomly choose the state to which one transitions after seeing a 0 and the state to which one transitions after seeing a 1. The fact that there is only one state to which one transitions after seeing a 0 or a 1 implies unifilarity of the resulting Hidden Markov model. The states of a minimal unifilar Hidden Markov model are called causal states [10].

We wish to understand how much memory is required to predict the output of these Hidden Markov models as well as possible and their predictability. Let ${\overset{\leftarrow }{X}}_{t}$ stand for the past environmental inputs, …, *X*_*t*−2_, *X*_*t*−1_, *X*_*t*_. The memory required is characterized by statistical complexity $C_{\mu }=H\left[S_{t}\right]$ [10], while the predictability is characterized by the total correlation rate $\rho_{\mu }=I\left[{\overset{\leftarrow }{X}}_{t};X_{t+1}\right]$ [11] also known as a particular value of the predictive information [12, 13].

As the underlying model grows larger, *ρ*_*μ*_ seems to tend in probability to ∼ 0.2 nats. These unifilar Hidden Markov models generate infinite-order Markov processes– that is, a process for which the next symbol depends to some extent on all previous symbols. However, a process that is technically infinite-order Markov can still be approximately Markovian [14]. A sensor which perfectly stores the present observed symbol and nothing else (a Markov model) would capture a predictive information of *I*[*X*_*t*_;*X*_*t*+1_]. A typical value of *I*[*X*_*t*_;*X*_*t*+1_] for these environments is 0.002 nats when the environment is of size |**S**| = 30, and so a typical value of *I*[*X*_*t*_;*X*_*t*+1_]/*ρ*_*μ*_ for these environments is 0.01. In other words, these environments tend to be strongly non-Markovian. And finally, the statistical complexity *C*_*μ*_ of these environments tends to be between 2 − 3 nats, indicating that these environments provide a predictive challenge for sensors.

### Model of sensor

Fix a realization of the environment, *x*_1_, *x*_2_, …. Let *R*_*t*_ be the random variable denoting the sensor’s state at time *t*. We consider conditionally Markovian sensors with a finite number of states and with state space *s* whose probability evolves according to

$$\begin{array}{c} Pr\left(R_{t+1}=r^{\prime }\right)=\sum_{r}Pr\left(R_{t+1}=r^{\prime }|X_{t}=x_{t},R_{t}=r\right)Pr\left(R_{t}=r\right), \end{array}$$ (4)

which we represent in matrix-vector notation as

$$\begin{array}{c} p\left(r_{t+1}\right)=M^{\left(x_{t}\right)}p\left(r_{t}\right). \end{array}$$ (5)

**Fig 1:**
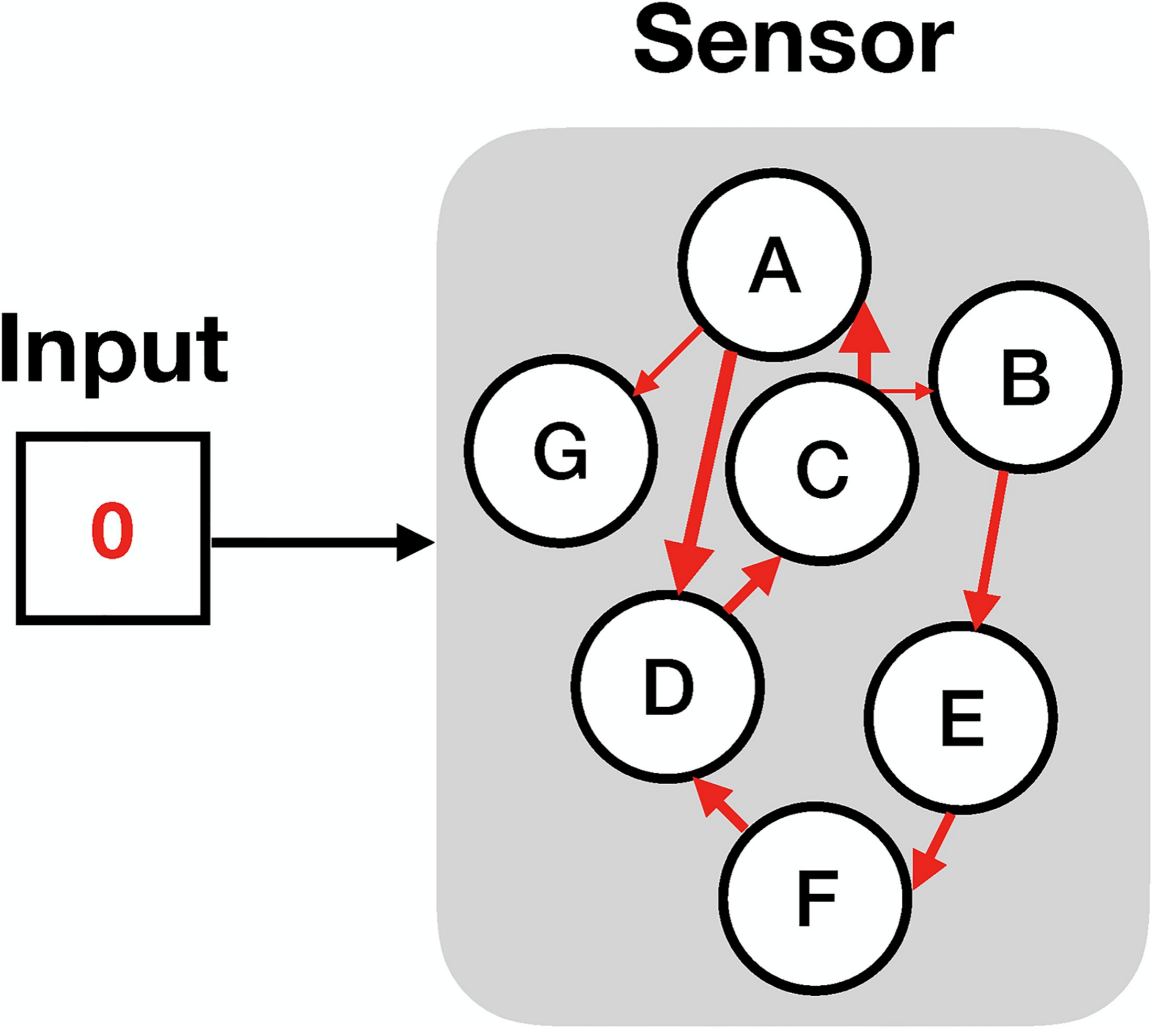
The model of the sensor. The input here is binary– either 0 or 1– and the sensor states comprise the set {*A*, *B*, *C*, *D*, *E*, *F*, *G*}. Different environmental inputs trigger different transition probabilities between the states of the sensor. These transition probabilities can be large (fat arrows) or small (thin arrows). The sensor shown here is nearly deterministic given an input of 0, in that each sensor state only transitions to either one or two other sensor states.

Given its relation to the conditional probability distribution, *M*^(*x*_*t*_)^ is a transition matrix whose columns must sum to 1. See Fig 1, in which the arrows between sensor states indicate transition probabilities and in which the arrow from input to sensor states indicates a dependence of transition probabilities on input.

The present sensor state depends on both the previous environmental symbol and the previous sensor state, and the recursion leads to the present sensor state depending on arbitrarily long environmental pasts. By construction, the sensor only understands the future of the environment through the past of the environment. In other words, the Markov relation $R_{t}\to {\overset{\leftarrow }{X}}_{t}\to X_{t+1}$ holds [9], meaning that $Pr\left(X_{t+1}=x,R_{t}=r|{\overset{\leftarrow }{X}}_{t}=\overset{\leftarrow }{x}\right)=Pr\left(X_{t+1}=x|{\overset{\leftarrow }{X}}_{t}=\overset{\leftarrow }{x}\right)Pr\left(R_{t}=r|{\overset{\leftarrow }{X}}_{t}=\overset{\leftarrow }{x}\right)$.

### Sensor metrics: Memory and predictive information

We define memory *I*_*mem*_ as

$$\begin{array}{c} I_{mem}:=I\left[R_{t};S_{t}\right], \end{array}$$ (6)

which is an achievable minimal coding cost of pasts– that is, the minimal amount of space needed to write down information about said pasts– to retain information about the future [15]. In terms of the steady-state probability distribution over input causal states and sensor states *p*_*ss*_(*r*, *σ*), we have

$$\begin{array}{c} I_{mem}=\sum_{r,\sigma }p_{ss}\left(r,\sigma \right)\log\frac{p_{ss}\left(r,\sigma \right)}{p_{ss}\left(r\right)p_{ss}\left(\sigma \right)}, \end{array}$$ (7)

where *p*_*ss*_(*r*) = ∑_*σ*_ *p*_*ss*_(*r*, *σ*) and *p*_*ss*_(*σ*) = ∑_*r*_ *p*_*ss*_(*r*, *σ*). The memory *I*_*mem*_ is 0 only when *p*_*ss*_(*r*, *σ*) = *p*_*ss*_(*r*)*p*_*ss*_(*σ*)– that is, when the sensor is nonspecific for the predictive features of its input. Note, from a standard information theory identity *I*[*X*;*Y*] ≤ *H*[*X*], that

$$\begin{array}{c} I_{mem}\leq C_{\mu }, \end{array}$$ (8)

and so one’s memory is upper-bounded by the statistical complexity, which is calculable from *p*_*ss*_(*σ*).

We define instantaneous predictive information (which we call predictive information for brevity) *I*_*pred*_ as

$$\begin{array}{c} I_{pred}:=I\left[R_{t};X_{t+1}\right]. \end{array}$$ (9)

This predictive information corresponds (under some assumptions) to the increase in expected log growth rate of an asexually reproducing population attainable with a given memory, and is always an upper bound on the increase in expected log growth rate attainable with a given memory [16]. In terms of the steady-state distribution *p*_*ss*_(*r*, *x*) over sensor states and future inputs, we have

$$\begin{array}{c} I_{pred}=\sum_{r,x}p_{ss}\left(r,x\right)\log\frac{p_{ss}\left(r,x\right)}{p_{ss}\left(r\right)p_{ss}\left(x\right)}. \end{array}$$ (10)

Earlier, causality gave us $R_{t}\to {\overset{\leftarrow }{X}}_{t}\to X_{t+1}$; and as the hidden states of a unifilar Hidden Markov model are minimal sufficient statistics of prediction [10], we also have ${\overset{\leftarrow }{X}}_{t}\to S_{t}\to X_{t+1}$. Together, this gives $R_{t}\to S_{t}\to X_{t+1}$, and the Data Processing Inequality [9] therefore reveals

$$\begin{array}{c} I_{pred}\leq I_{mem}. \end{array}$$ (11)

That is, the predictive information captured is always less than one’s memory. Another application of the Data Processing Inequality reveals

$$\begin{array}{c} I_{pred}\leq \rho_{\mu }, \end{array}$$ (12)

and so the total correlation rate is an achievable upper bound on the predictive information. To achieve this upper bound, one needs the sensor states to uniquely determine all causal states *σ*.

We also define, for reasons that become apparent later on,

$$\begin{array}{c} I_{mem}^{\prime }:=D_{KL}\left[p_{ss}\left(r,\sigma \right)||\frac{1}{N}p_{ss}\left(\sigma \right)\right]. \end{array}$$ (13)

This is a measure of the deviation between the steady state distribution over sensor states and input causal states, *p*_*ss*_(*r*, *σ*), from the distribution over sensor states and input causal states were the sensor to be nonspecific for its input *and* were each sensor state to be equally likely. If the sensor is nonspecific for its input but if the distribution over sensor states is nonuniform, then $I_{mem}^{\prime }$ will be nonzero. As discussed in Section B in S1 File,

$$\begin{array}{c} I_{mem}\leq I_{mem}^{\prime }. \end{array}$$ (14)

As a result, if we can argue that $I_{mem}^{\prime }$ tends to 0, we will also have argued that *I*_*mem*_ and thus *I*_*pred*_ tend to 0.

## Methods

We wish to calculate the aforementioned sensor metrics. One could simulate sequences of *σ*_*t*_, *r*_*t*_, and *x*_*t*+1_ by using *T*^(*x*)^, *M*^(*x*)^ to randomly choose future sensor and environmental states, and then count the number of occurrences of a particular combination of *σ*_*t*_, *r*_*t*_, *x*_*t*+1_. In the case of a large sensor state space, one would ideally use an entropy estimator such as the NSB entropy estimator [17] to calculate the predictive information, so as to avoid prohibitively long simulations.

However, we pursue a different approach that leads to an easier calculation of sensor metrics and to plausibility arguments that underscore the generality of our results. To calculate *I*_*mem*_, we wish to find *p*(*r*_*t*_, *σ*_*t*_). To calculate instantaneous predictive information *I*_*pred*_, we wish to find *p*(*r*_*t*_, *x*_*t*+1_). We can get the latter probability distribution, *p*(*r*_*t*_, *x*_*t*+1_), from the former probability distribution, *p*(*r*_*t*_, *σ*_*t*_), by exploiting the Markov chain $R_{t}\to S_{t}\to X_{t+1}$:

$$\begin{array}{ccc} p(r_{t},x_{t+1}) & = & \sum_{\sigma_{t}}p\left(r_{t},\sigma_{t},x_{t+1}\right) \end{array}$$ (15)

$$\begin{array}{ccc} & = & \sum_{\sigma_{t}}p\left(r_{t},\sigma_{t}\right)p\left(x_{t+1}|\sigma_{t}\right). \end{array}$$ (16)

To find *p*(*r*_*t*_, *σ*_*t*_), we can set up a Chapman-Kolmogorov equation:

$$\begin{array}{ccc} p(r_{t+1},\sigma_{t+1}) & = & \sum_{r_{t},\sigma_{t},x_{t}}p\left(r_{t+1},\sigma_{t+1}|r_{t},\sigma_{t},x_{t}\right)\\ & & \times p(r_{t},\sigma_{t},x_{t}) \end{array}$$ (17)

$$\begin{array}{ccc} & = & \sum_{r_{t},\sigma_{t},x_{t}}p\left(r_{t+1}|x_{t},r_{t}\right)p\left(\sigma_{t+1}|x_{t},\sigma_{t}\right)p\left(x_{t}|\sigma_{t}\right)\\ & & \times p(r_{t},\sigma_{t}) \end{array}$$ (18)

$$\begin{array}{ccc} & = & \sum_{r_{t},\sigma_{t},x_{t}}M_{r_{t+1},r_{t}}^{\left(x_{t}\right)}\frac{T_{\sigma_{t+1},\sigma_{t}}^{\left(x_{t}\right)}}{1^{⊤}T_{:,\sigma_{t}}^{\left(x_{t}\right)}}(1^{⊤}T_{:,\sigma_{t}}^{\left(x_{t}\right)})\\ & & \times p(r_{t},\sigma_{t}) \end{array}$$ (19)

$$\begin{array}{ccc} & = & \sum_{r_{t},\sigma_{t}}(\sum_{x_{t}}M_{r_{t+1},r_{t}}^{\left(x_{t}\right)}T_{\sigma_{t+1},\sigma_{t}}^{\left(x_{t}\right)})p\left(r_{t},\sigma_{t}\right). \end{array}$$ (20)

Eq 20 defines a vector with |*R*||**S**| elements and a corresponding transition matrix. The normalized eigenvector of eigenvalue 1 corresponds to the desired *p*_*ss*_(*r*, *σ*), and then one can calculate the instantaneous predictive information directly from *p*(*r*_*t*_, *x*_*t*+1_) via

$$\begin{array}{c} I_{pred}=\sum_{r_{t},x_{t+1}}p\left(r_{t},x_{t+1}\right)\log\frac{p(r_{t},x_{t+1})}{p\left(r_{t}\right)p\left(x_{t+1}\right)}. \end{array}$$ (21)

This serves as an alternative to calculating *p*_*ss*_(*r*, *σ*), and thus *I*_*mem*_ and *I*_*pred*_, through simulation.

## Results

There are two parameters that one can play with when designing our randomly wired sensors: sensor size, given by the number of sensor states *N*; and the method by which connections between sensor states are randomly generated.

Using a sensor of size *N* corresponds to clustering pasts into *N* clusters: an input past $\overset{\leftarrow }{x}$ leads to sensor state *r* with probability $p(r|\overset{\leftarrow }{x})$. When the sensor is not deterministic, these clusters are *soft clusters*– that is, pasts are assigned probabilistically to clusters. When sensor connections are closer to deterministic, these clusters “harden”. From Section A in S1 File, we then expect the memory and predictive information captured by sensors to increase (but saturate) with increasing sensor size and increasing determinism.

In other words, specification of a randomly wired sensor corresponds to a random soft clustering of pasts into predictive features, though the mapping from the sensor specification to the clustering of pasts is nontrivial. Despite the presence of such a mapping, specification of a sensor has one important advantage over specification of a probabilistic clustering of pasts: it is a finite description of a potentially complicated clustering. More concretely, it is more economical to specify the optimal predictor (the *ϵ*M [10]) by input-dependent state transitions, *M*^(*x*)^, instead of the conditional probability distribution of hidden states given input histories, $p(r|\overset{\leftarrow }{x})$.

**Fig 2:**
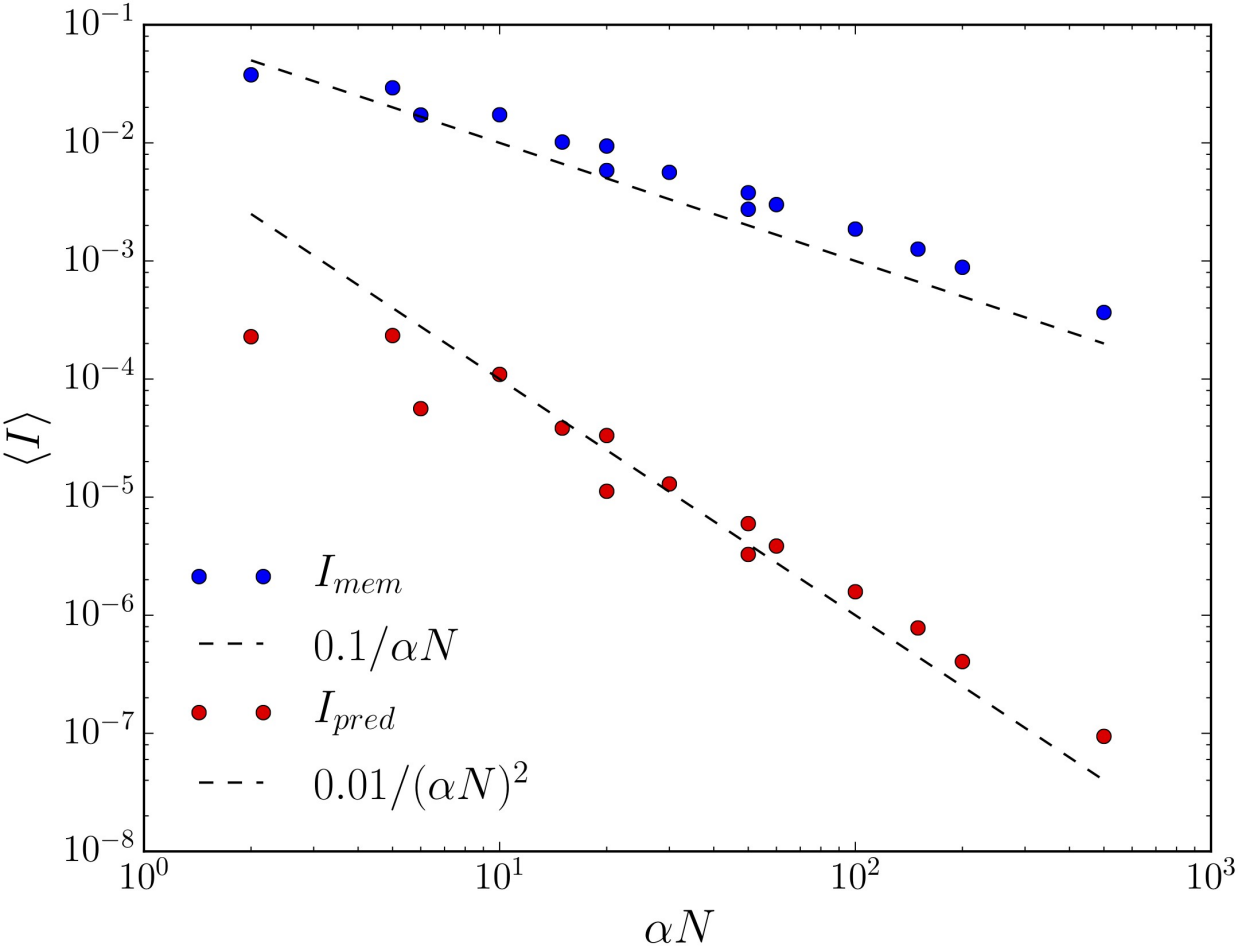
Large, fully nondeterministic, randomly wired sensors are nonspecific for their inputs. As described in the main text, the concentration parameter *α* controls the distribution from which transition probabilities in the sensor are drawn. We show 〈*I*_*mem*_〉 (blue) and 〈*I*_*pred*_〉 (red) for three values of *α*– 1, 3, and 10– as a function of *αN*. Both information quantities decrease at varying rates with *N* and *α*: roughly 0.1/*αN* for 〈*I*_*mem*_〉, and roughly 0.01/(*αN*)^2^ for 〈*I*_*pred*_〉. Corresponding lines in black dashes are drawn to guide the eye. The environment has total correlation rate *ρ*_*μ*_ = 0.198 nats and statistical complexity *C*_*μ*_ = 2.205 nats, so the total memory and predictive information captured by the sensor is maximally three orders of magnitude smaller than the total possible memory and predictive information captured.

First, we consider fully nondeterministic, randomly wired sensors for which the columns of *M*^(*x*)^ are independent and identically distributed (i.i.d) draws from a Dirichlet distribution with concentration parameter $\vec{\alpha }=\alpha \vec{1}$. We start with a strange numerical fact. As such sensors grow in size, when *α* is sufficiently large, both memory and predictive information tend to decrease. See Fig 2. In fact, as *N* grows, *p*(*r*|*σ*) appears to tend to $\frac{1}{N}$ – that is, the sensor appears to become nonspecific for its input. In other words, the capacity of a sensor to remember its input increases as the sensor increases in size, but this capacity is not at all used by fully connected, randomly wired sensors.

We give a plausibility argument for this nonspecificity, which comes down to a statement about the eigenvector of eigenvalue 1 of the transition matrix between sets of causal states and sensor states, (*σ*,*r*), with elements $\sum_{x_{t}}M_{r_{t+1},r_{t}}^{\left(x_{t}\right)}T_{\sigma_{t+1},\sigma_{t}}^{\left(x_{t}\right)}$ as described in Methods. This argument is generalized and expanded upon in Section B in S1 File. There is a unique eigenvector of eigenvalue 1 for these transition matrices by the Perron-Frobenius theorem. We will argue that this eigenvector is given in the large *N* limit by $p\left(\sigma ,r\right)=\frac{1}{N}p_{ss}\left(\sigma \right)$ where *p*_*ss*_(*σ*) = eig_1_(∑_*x*_ *T*^(*x*)^)_*σ*_. If $p\left(\sigma ,r\right)=\frac{1}{N}p_{ss}\left(\sigma \right)$, then

$$\begin{array}{ccc} \frac{1}{N}p_{ss}\left(\sigma_{t+1}\right) & = & \sum_{\sigma_{t},x_{t}}(\frac{1}{N}\sum_{r_{t}}M_{r_{t+1},r_{t}}^{\left(x_{t}\right)})T_{\sigma_{t+1},\sigma_{t}}^{\left(x_{t}\right)}p_{ss}\left(\sigma_{t}\right). \end{array}$$

To understand whether or not this is plausible, we focus on simplifying the right-hand side of this equation. Recall that a Dirichlet distribution in which the concentration parameter vector takes the form $\vec{\alpha }=\alpha \vec{1}$ can be generated by drawing realizations of identical and independently distributed (i.i.d.) Gamma random variables and normalizing them by their sum. Hence, we can write $M_{r_{t+1},r_{t}}^{\left(x_{t}\right)}=\frac{Y_{r_{t+1},r_{t}}^{\left(x_{t}\right)}}{\sum_{r^{\prime }}Y_{r^{\prime },r_{t}}^{\left(x_{t}\right)}}$ where $Y_{r^{\prime },r_{t}}^{\left(x_{t}\right)}$ have been drawn i.i.d. from a distribution with probability density function $\frac{1}{\Gamma (\alpha )}y^{\alpha -1}e^{-y}$. Then, the sum $\sum_{r_{t}}M_{r_{t+1},r_{t}}^{\left(x_{t}\right)}=\sum_{r_{t}}\frac{Y_{r_{t+1},r_{t}}^{\left(x_{t}\right)}}{\sum_{r^{\prime }}Y_{r^{\prime },r_{t}}^{\left(x_{t}\right)}}$ is the ratio of two roughly independent Gamma distributions, both with shape parameter *Nα* and scale 1. The ratio of two such Gamma distributions is highly peaked at 1 for large *Nα*. Thus $\sum_{r_{t}}M_{r_{t+1},r_{t}}^{\left(x_{t}\right)}$ tends to 1, which then implies that

$$\begin{array}{ccc} \sum_{\sigma_{t},x_{t}}(\frac{1}{N}\sum_{r_{t}}M_{r_{t+1},r_{t}}^{\left(x_{t}\right)})T_{\sigma_{t+1},\sigma_{t}}^{\left(x_{t}\right)}p_{ss}\left(\sigma_{t}\right) & \to & \frac{1}{N}p_{ss}\left(\sigma_{t+1}\right) \end{array}$$

as desired, using $\sum_{\sigma_{t}}T_{\sigma_{t+1},\sigma_{t}}^{\left(x_{t}\right)}p_{ss}\left(\sigma_{t}\right)=p_{ss}\left(\sigma_{t+1}\right)$. In other words, when *Nα* ≫ 1, $p\left(r|\sigma \right)=\frac{1}{N}$ appears to be a reasonable guess for the steady state distribution *p*_*ss*_(*r*|*σ*). This, in turn, suggests that *I*_*mem*_ tends to 0 with probability 1, which would give *I*_*pred*_ → 0 from 0 ≤ *I*_*pred*_ ≤ *I*_*mem*_. An additional argument given in Section B in S1 File suggests that $I_{mem}^{\prime }$ is $O(\frac{1}{N\alpha })$, though we emphasize that this is a plausibility argument rather than a sketch of a proof.

**Fig 3:**
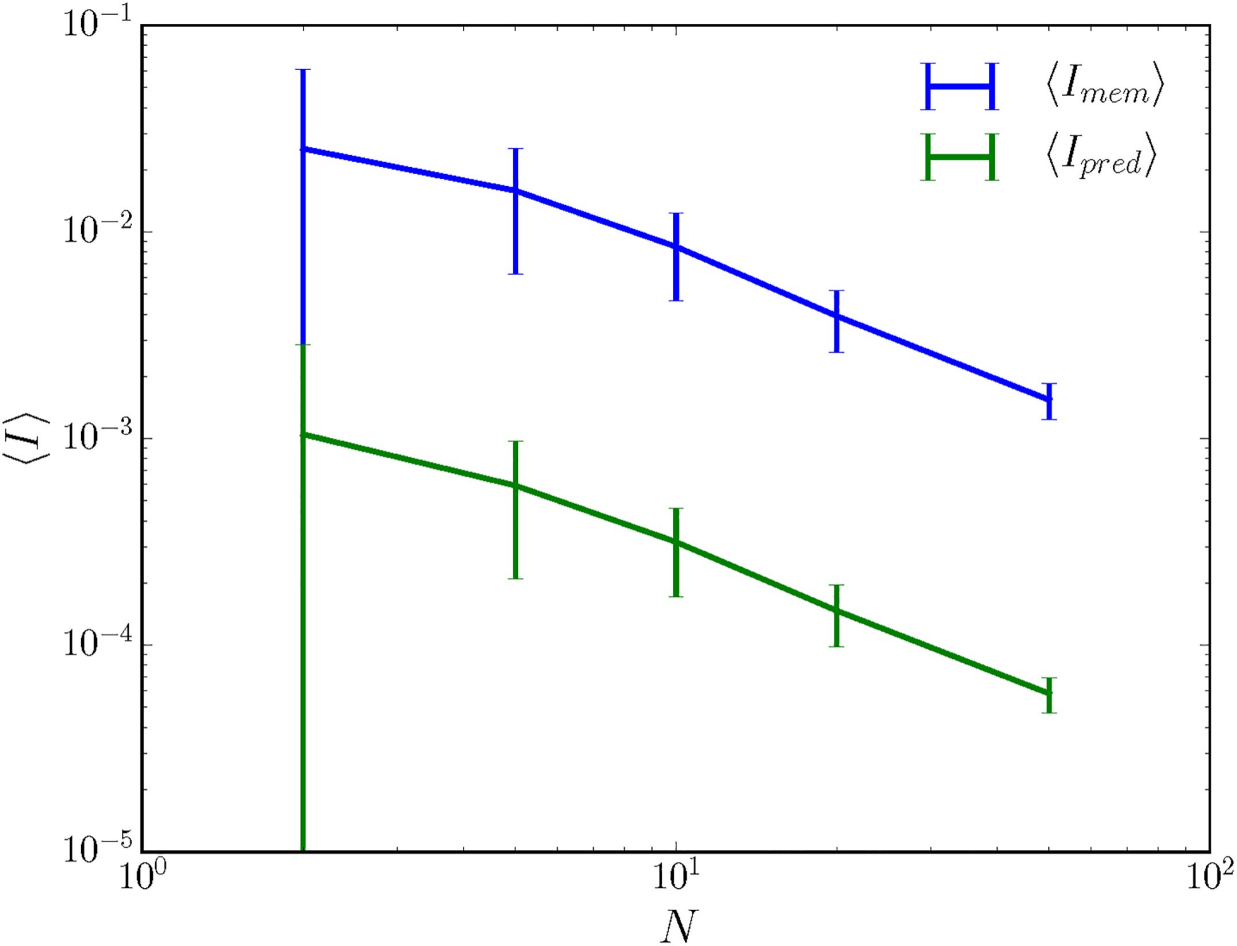
Large, nondeterministic, randomly wired sensors are nonspecific for their inputs even when the stochasticity is input- and state-dependent. As described in the main text, the concentration parameter $\vec{\alpha }$ describes the random wiring of the sensors. Here, *α*(*x*, *r*) is drawn uniformly at random in the interval [0, 10] for each *x*. Both average memory and average predictive information decrease with sensor size *N*. The environment again has |**S**| = 30 but with *ρ*_*μ*_ = 0.19 nats and *C*_*μ*_ = 3.1 nats. The 50% confidence intervals in memory and predictive information decrease with increasing sensor size *N*, implying that the larger sensors are increasingly likely to be nonspecific for their input.

In other words, infinitely large fully-connected randomly-wired sensors are nonspecific for their inputs, *no matter the input*. This is true even when concentration parameter $\vec{\alpha }$ defining the sensor stochasticity is input-dependent and state-dependent, as an extension of the plausibility argument given above holds as detailed in Section B in S1 File. For numerical evidence, see Fig 3.

**Fig 4:**
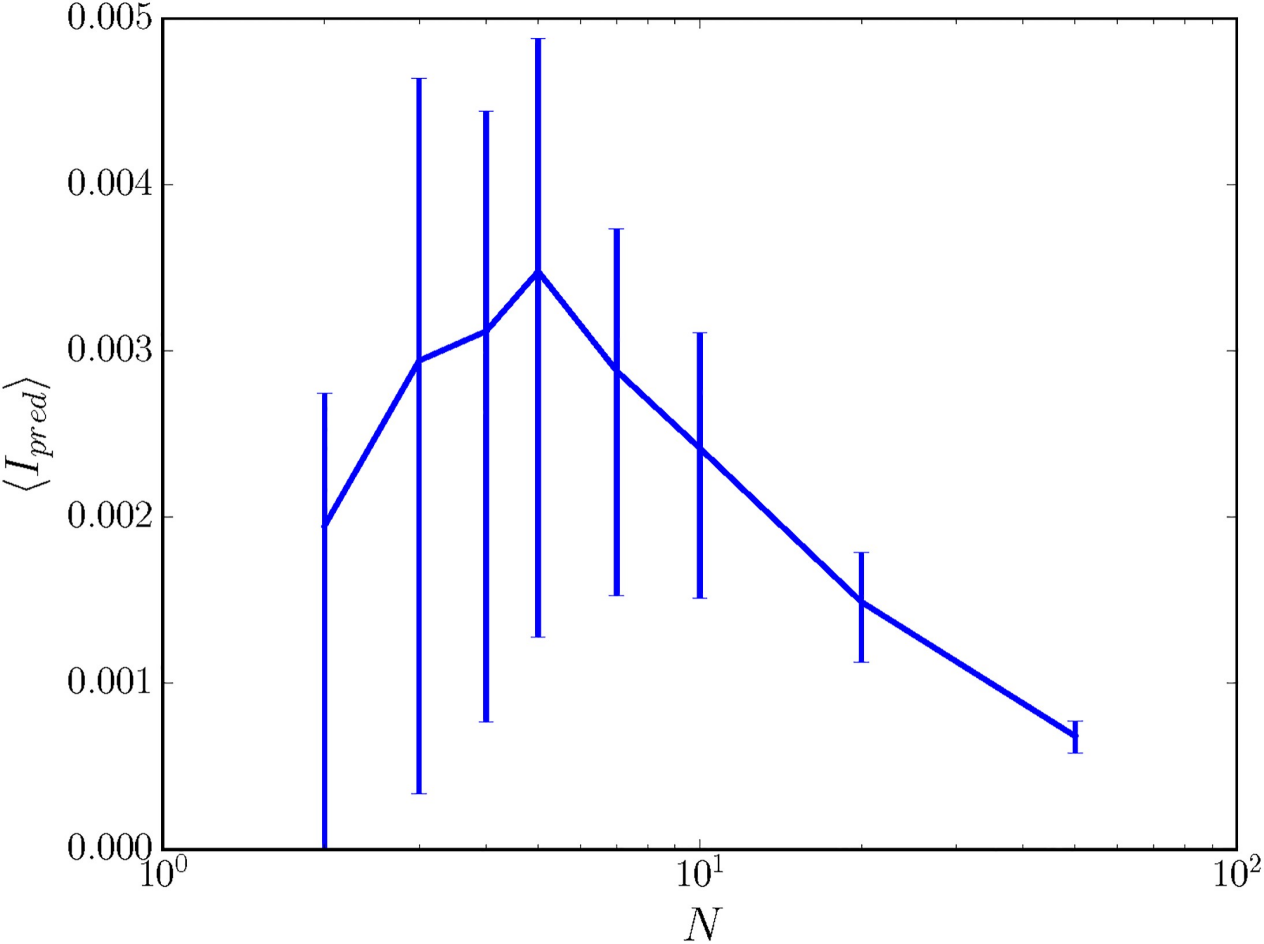
Predictive information can increase or decrease with sensor size. We show average predictive information 〈*I*_*pred*_〉 as a function of *N* for *α* = 0.1, where 〈*I*_*pred*_〉 is estimated from 100 random draws of the sensor. The environment has |**S**| = 30, and the non-monotonicity of 〈*I*_*pred*_〉 with *N* for this value of *α* seems to hold regardless of environment. Error bars indicate 50% confidence intervals.

More generally, we find that the memory and predictive information captured by random sensors is governed by two trends. Both trends can be described in terms of their effect on the clusters $p(r|\overset{\leftarrow }{x})$ that describe how specifically one can determine an input past $\overset{\leftarrow }{x}$ from a sensor state *r* and vice versa. According to the first trend, information captured tends to increase with the number of clusters, as detailed by a null model in Section A in S1 File. According to the second trend, the exponential explosion in possible paths between sensor states given any particular input past $\overset{\leftarrow }{x}$ yields increasing nonspecificity. For small enough *α*, as shown in Fig 4, the behavior of memory and predictive information captured by randomly wired sensors appears to be a balance of these two trends. However, the latter trend for fully nondeterministic sensors always wins, and so infinitely large, fully nondeterministic, randomly wired sensors are completely nonspecific for their inputs even though the sensor dynamics are input-dependent.

How might a large system with unavoidable randomness in its connections avoid the seemingly inevitable march towards nonspecificity for its inputs? After all, large sensors have a large capacity to predict, in principle, harnessed in Refs. [3, 4]. Is there no way in which randomness in wiring can be constrained so that this capacity can be harvested?

A clue is provided by consideration of the minimal optimal predictive sensor of the inputs [10]– the *ϵ*M, of size N=|R|=|S|. This optimal sensor is constructed so that each *r* corresponds to a different causal state *σ*, with transitions $M_{r,r^{\prime }}^{\left(x\right)}=Pr\left(S_{t+1}=r^{\prime }|X_{t}=x,S_{t}=r\right)$. (When $Pr(X_{t}=x|S_{t}=r)$ is zero, a particular input word is forbidden, and any $M_{r,r^{\prime }}^{\left(x\right)}$ can be chosen.) Note that for this optimally predictive sensor, given a particular input *x*, a particular sensor state *r* can only transition to one other sensor state *r*′. Minimal optimal predictive sensors, therefore, have a great deal of structure: they are deterministic. Many transitions between sensor states are forbidden.

Indeed, sensors optimized so as to maximize predictive information using the L-BFGS algorithm are also nearly deterministic. These (locally) optimal sensors tend to make Markov models of the input, capturing 0.04 nats or 20% of the total predictive information capturable with |R|=10 states.

**Fig 5:**
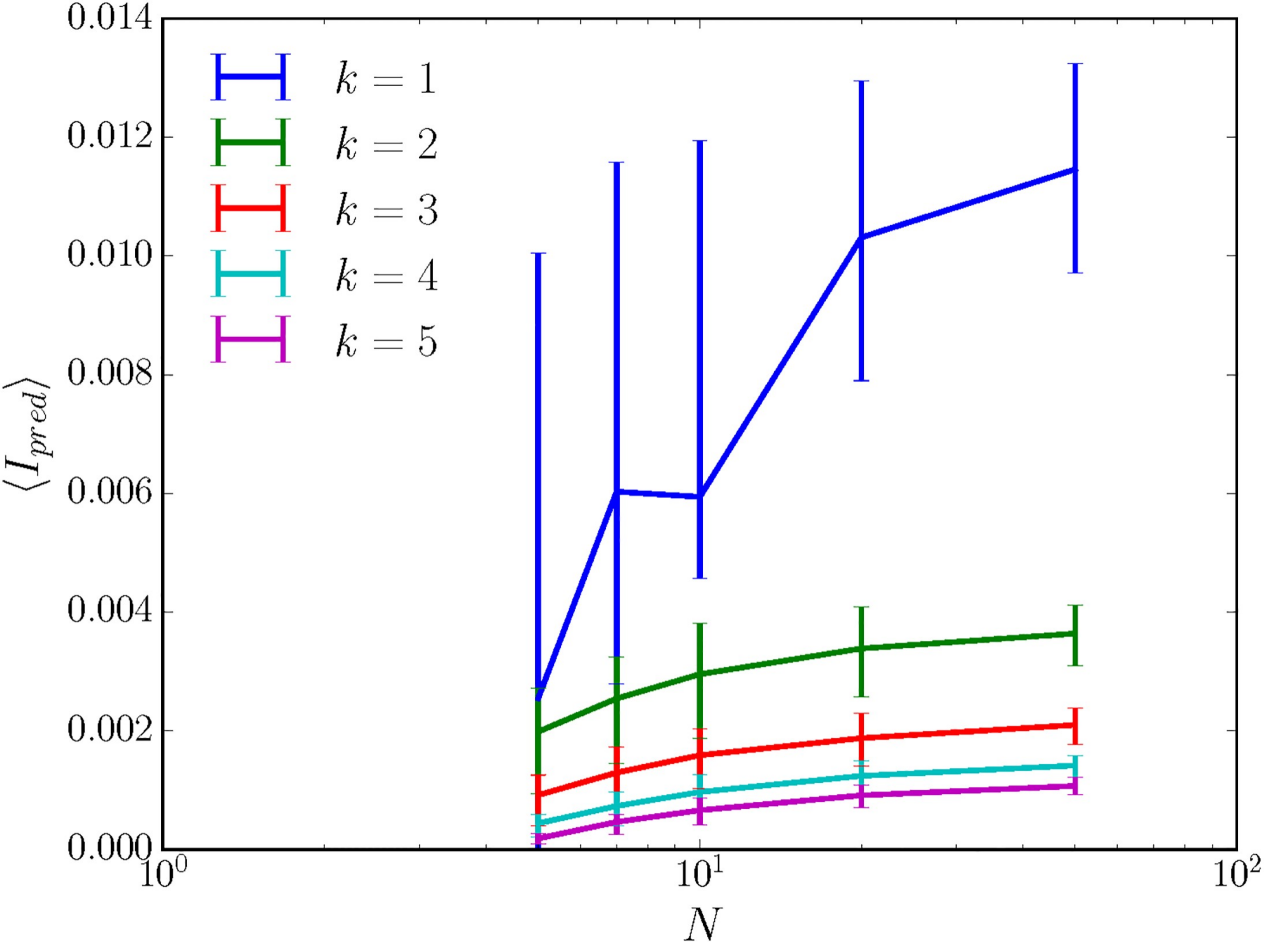
Nearly deterministic, randomly wired sensors capture more predictive information than nondeterministic randomly wired sensors. Nearly deterministic sensors are randomly generated as described in the main text with *α* = 3, *k* as given in the legend, and *N* as given by the *x*-axis. 100 sensors are generated at each possible sensor size N=|R|, and their predictive informations *I*_*pred*_ are averaged to give 〈*I*_*pred*_〉. 50% confidence intervals in 〈*I*_*pred*_〉 are given by the error bars. The environment has |**S**| = 30, *ρ*_*μ*_ = 0.23 nats, and *C*_*μ*_ = 2.3 nats, and so these randomly-wired sensors are still only capturing ∼ 10% of the total predictive information possible.

**Fig 6:**
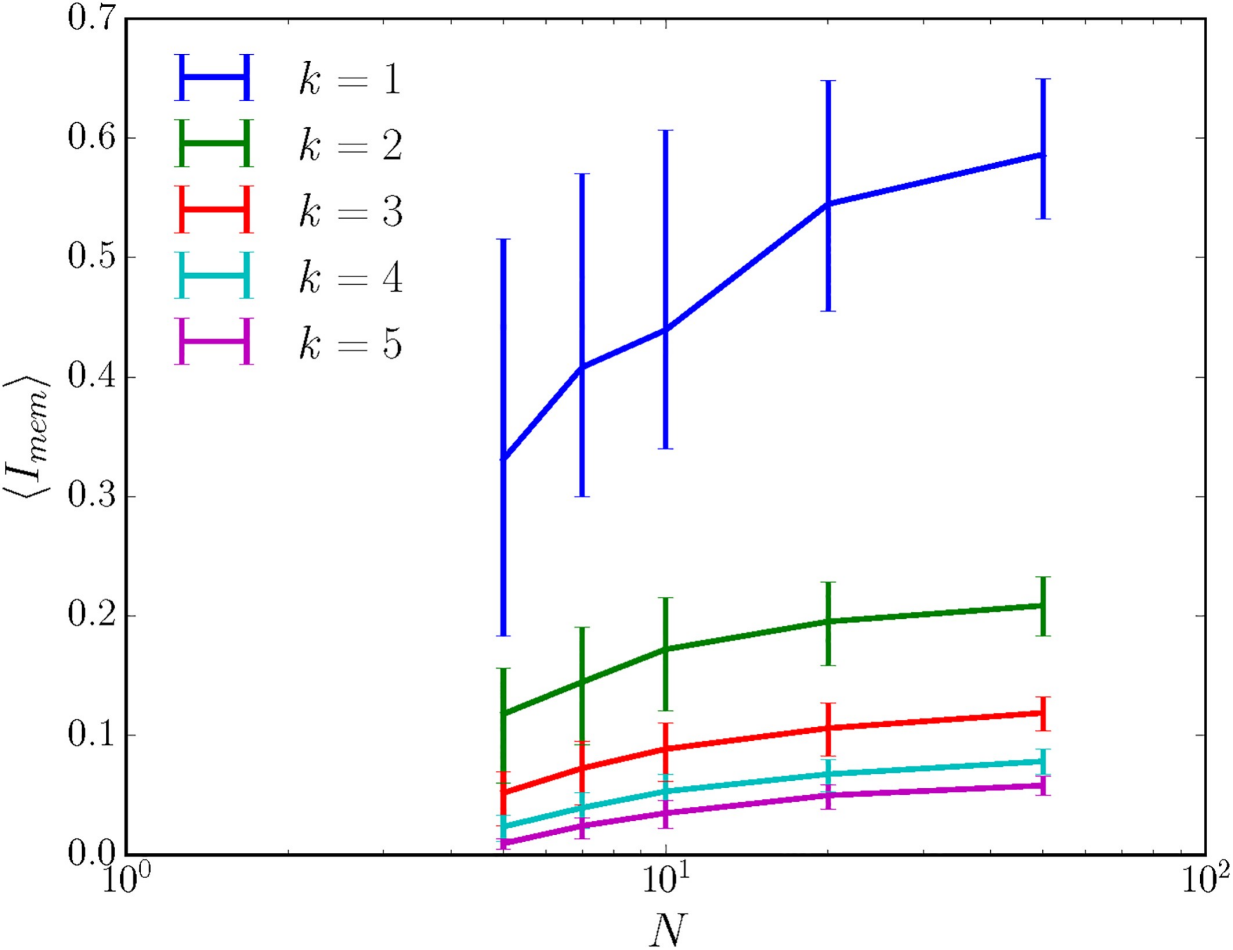
Nearly deterministic, randomly wired sensors have more memory than nondeterministic randomly wired sensors. Nearly deterministic sensors are randomly generated as described in the main text with *α* = 3, *k* as given in the legend, and *N* as given by the *x*-axis. 100 sensors are generated at each possible sensor size N=|R|, and their predictive informations *I*_*mem*_ are averaged to give 〈*I*_*mem*_〉. 50% confidence intervals in 〈*I*_*mem*_〉 are given by the error bars. The environment has |**S**| = 30, *ρ*_*μ*_ = 0.23 nats, and *C*_*μ*_ = 2.3 nats, and so these randomly-wired sensors are only capturing ∼ 25% of the total memory possible.

Perhaps unsurprisingly, then, large randomly wired sensors turn out to be specific for their inputs when the sensor is very close to deterministic. Then, $\sum_{r_{t}}M_{r_{t+1},r_{t}}^{\left(x_{t}\right)}$ will not be highly concentrated about 1, and the plausibility arguments given above for nonspecificity will fail. To illustrate this, we consider the case of a sensor in which $M_{r,r^{\prime }}^{\left(x\right)}$ is, for each initial sensor state *r*′, nonzero only for *k* randomly drawn sensor states *r*; and in which the probability distribution over the *k* nonzero $M_{r,r^{\prime }}^{\left(x\right)}$ is Dirichlet with concentration parameter *α*. We see from Fig 5 that the greater the determinism (i.e. the smaller the *k*), the higher the average predictive information; and that, no matter the *k*, the predictive information captured actually increases with the size of the sensor. Large, nearly deterministic, randomly wired sensors have variable values of predictive information, unlike the large, fully nondeterministic, randomly wired sensors as discussed in Section C in S1 File. The average predictive information captured appears to saturate with increasing sensor size, and as such, the quality of the sensor grows with *N* but is fundamentally limited by *k*. The same trends hold for average memory 〈*I*_*mem*_〉, shown in Fig 6.

Note, however, that if *k* grows with *N* such that lim_*N*→∞_ *k* is infinite, then the plausibility arguments given before will apply and the infinitely large, randomly wired sensor will be nonspecific for its input. Hence, the sparsity of sensor connections– the number of connections divided by the number of possible connections– must tend to 0 as the sensor size increases if an infinitely large randomly-wired sensor is to be at all specific for its input.

## Conclusion

Evolution and scientists have a difficult task: that of creating predictive sensors of time series input that may contain long-range correlations. One can approach this problem by designing learning rules that adjust sensor connection weights so as to optimize sensor predictive capabilities. Nondeterministic sensors can result. Alternatively, one can approach this problem by randomly wiring a sensor and optimizing the sensor readout. We have shown that success of the latter approach requires determinism or something close to it, in that given a present sensor state and present input, one should be able to transition to only a few sensor states in the next time step.

One way to conceptualize the sensor’s predictive computation is to view each sensor state as a cluster of pasts– that is, given a particular input past $\overset{\leftarrow }{x}$, there is a probability $p(r|\overset{\leftarrow }{x})$ of ending up in sensor state *r* determined in some nonlinear fashion by the dynamics of the input and the dynamics of the sensor. When a sensor is nondeterministic, these clusters are soft clusters, meaning that a number of sensor states *r* are possible given a particular input past $\overset{\leftarrow }{x}$. More sensor states correspond to more clusters, which as shown in Section A in S1 File, tends to increase memory and predictive information captured. However, an increase in the number of sensor states provides one with exponentially more pathways from one sensor state to the next for a particular input past, which on average have equivalent total weights for each input past. These two competing trends can lead to a nonmonotonic dependence of memory and predictive information on sensor size. At large enough sensor sizes, the latter trend always dominates. Hence, infinitely large, fully nondeterministic, randomly wired sensors are increasingly nonspecific for their input. To break the trend towards nonspecificity, one needs to restrict the number of possible paths between sensor states with determinism.

One may well ask what we have learned about optimal sensors from the model presented here. After all, it is well-known in signal estimation problems that sensor stochasticity is detrimental. The interesting twist in our story is that when discussing *recurrent* sensors, the detrimental effects of sensor stochasticity are compounded rather than mitigated by increasing sensor size. Feedforward sensors correspond to the null model studied in Section A in S1 File, while recurrent sensors correspond to the eigenvector analysis of the main text.

In essence, we have asked what kinds of random sensor ensembles yield greater predictive power. This is certainly not the first time that such a question has been asked, e.g. Refs. [7, 18, 19]. Previously studied reservoirs were all deterministic– or rather, the variable that evolved in a conditionally Markovian manner (e.g., membrane voltages as opposed to neural activities) in the reservoir computing applications evolved deterministically. As such, our report on the extremely detrimental effects of nondeterminism on recurrent sensors is new, if perhaps unsurprising. When sensors are deterministic, one can study the effect of the spectral radius of the weight matrix used to evolve the sensor state, the sparsity of aforementioned weight matrix, the function applied to the weight matrix multiplied by the sensor state, and sensor size (as studied here), among other things.

True determinism in physical systems is impossible [20, 21]. Sometimes, noise can be beneficial to the functioning of biological systems [16, 20, 22, 23], but our work here suggests that this noise must be tightly controlled when one wants to remember or predict input. For instance, in a chemical reaction network, too many different possible reactions for a given environmental forcing (nondeterminism) will lead to a nonspecific response, e.g. sparsity in random reaction networks was key to the results of Ref. [24]. Our results suggest that given a particular inherent sensor stochasticity, there is an optimal finite sensor size at which functionality (prediction) is maximized. The size of sensors in biological systems, then, might not always be governed by resource constraints [25–27] but instead governed by degradation of functionality due to unavoidable noise.

## Supporting information

S1 File Section A: Random soft clusters in the information bottleneck method. Section B: Plausibility argument for nonspecificity of large randomly-wired sensors. Section C: Fluctuations in memory and prediction.
(PDF)

S1 Fig A null model for the effect of sensor size on predictive power predicts that larger sensors capture more information. The average information obtained about the relevant variable *Y*. The various lines correspond to various values of *α*, as indicated in the legends, and the *x*-axis corresponds to variation in the number of clusters *N*. We chose *M* = 30.
(TIFF)

S2 FigVariability in predictive information decreases with sensor size for fully nondeterministic sensors.On the *x*-axis is |R|, or *N*, and on the *y*-axis is the interquartile range (IQR) of *I*_*pred*_. The environment has *ρ*_*μ*_ = 0.147 nats and *C*_*μ*_ = 2.36 nats, but these results seemed to hold qualitatively regardless of particular environment.(TIFF)
