# Supplementary material for: Infinitely large, randomly wired sensors cannot predict their input unless they are close to deterministic
Source: PLoS One. 2018 Aug 29;13(8):e0202333. doi: 10.1371/journal.pone.0202333 (PMC6114800; doi:10.1371/journal.pone.0202333)
Supplement: S1 File — Section A: Random soft clusters in the information bottleneck method. Section B: Plausibility argument for nonspecificity of large randomly-wired sensors. Section C: Fluctuations in memory and prediction. (PDF) [file pone.0202333.s001.pdf]

Supporting information for "Infinitely large, randomly wired sensors cannot predict their input unless they are close to deterministic"

Sarah E. Marzen[1*]

1 Department of Physics, Physics of Living Systems Group, Massachusetts Institute of Technology, Cambridge, MA, USA

* semarzen@mit.edu

A Random soft clusters in the information bottleneck method

Earlier, we related specification of a sensor to a random clustering of pasts, in which the number of clusters was given by the sensor size and in which the softness of clusters was controlled by the concentration parameter α. This suggests a simple null model with which to benchmark our study of randomly wired predictive sensors: that of a random soft clustering R of some random variable X to retain information about another random variable Y [1]. For instance, to more explicitly draw the connection between this null model and the situation studied in the main text, the variable to be clustered might be forward-time causal states, and the relevant random variable might be the future observed symbol.

The joint probability distribution $p(x, y)$ must first be given. For the sake of simplicity, we assume a uniform distribution $p(x) = 1/M$, $M = |\mathcal{X}| = 30$, and $p(y|x)$ is drawn from a Dirichlet distribution with a concentration parameter of 1. The qualitative results were invariant to the particular draw of $p(y|x)$. The alphabet size of Y is $|\mathcal{Y}| = 2$. The choices of $M = 30$ and $|\mathcal{Y}| = 2$ were made so that X was analogous to forward-time causal states and Y was analogous to the future observed symbol.

Finally, we draw $p(r|x)$ randomly from a Dirichlet distribution with concentration parameter α over N states, with $|\mathcal{R}| = N$. Values of $I[X; R]$ and $I[R; Y]$ are straightforwardly computed from $p(x, r) = p(r|x)p(x)$ and $p(r, y) = \sum_x p(x, y)p(r|x)$ for each of 100 runs, and then averaged. The quantity $I[X; R]$ corresponds to I_{mem}; the quantity $I[R; Y]$ corresponds to I_{pred}; and the averages $\langle\cdot\rangle$ denote the average over 100 different draws of $p(r|x)$.

The results of the averaging are shown in Fig. 1. The graph shows that the analogue of I_{pred}, $I[R; Y]$, decreases with increasing α (increasing cluster softness) and increases with increasing N (number of clusters). Similar statements hold for $I[X; R]$, the analogue of I_{mem}. If these intuitions are imported directly over to the situation with randomly wired sensors, one would expect memory and predictive information to both increase with increasing cluster hardness, or decreasing α in the main text setup, and to both increase with increasing sensor size, or increasing N in the main text setup.

A closer analysis suggests that in fact, $I[X; R]$ and thus $I[R; Y]$ both saturate as $N,\ M \to \infty$. In the large N limit,

$$p(r) = \sum_x p(x)p(r|x) = \frac{1}{M}\sum_x p(r|x) \tag{1}$$

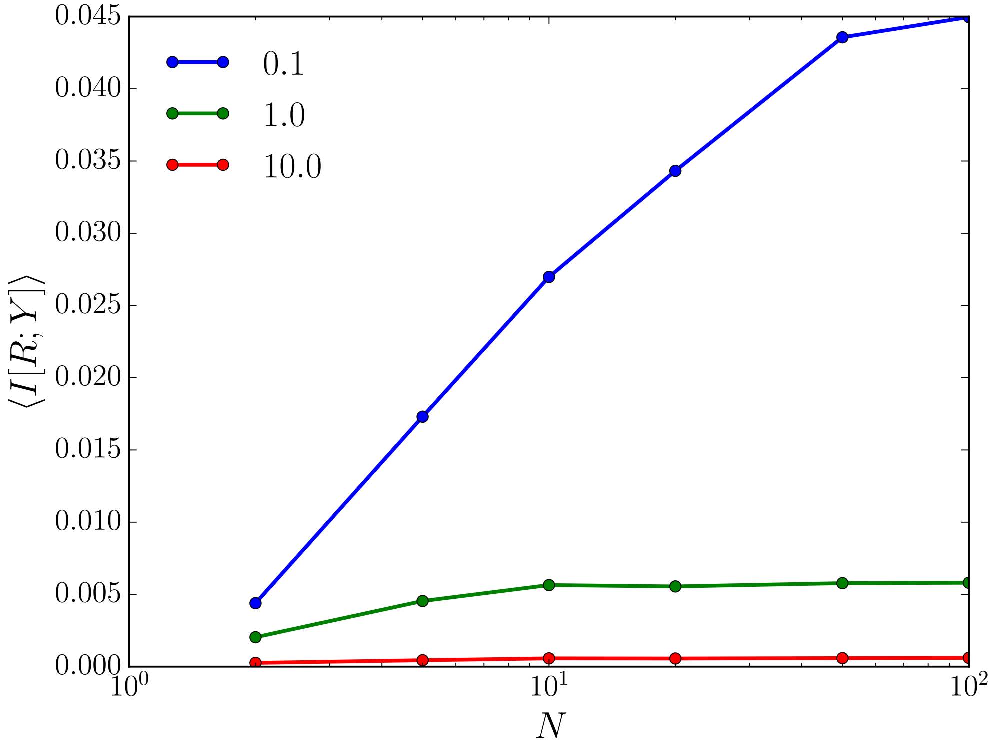

Fig 1. A null model for the effect of sensor size on predictive power predicts that larger sensors capture more information. The average information obtained about the relevant variable Y. The various lines correspond to various values of α, as indicated in the legends, and the x-axis corresponds to variation in the number of clusters N. We chose $M = 30$.

is a ratio of the sum of M identically and independently distributed Gamma random variables with shape parameter α and scale parameter 1 to a sum of N identically and independently distributed Gamma random variables with shape parameter α and scale parameter 1, with one random variable in common; and hence, $p(r)$ is $\frac{1}{M}$ times the ratio of a Gamma random variable with shape parameter $M\alpha$ and scale parameter 1 to a Gamma random variable with shape parameter $N\alpha$ and scale parameter 1, where in the large N limit, the numerator and denominator are essentially independent. Thus, $p(r)$ roughly has distribution

$$p(u) = \int_0^\infty \int_0^\infty \delta(u - \frac{1}{M}\frac{x}{y}) \frac{x^{M\alpha-1}e^{-x}}{\Gamma(M\alpha)} \frac{y^{N\alpha-1}e^{-y}}{\Gamma(N\alpha)} dxdy \quad (2)$$

$$= \frac{\Gamma((M+N)\alpha)}{\Gamma(M\alpha)\Gamma(N\alpha)} \frac{Mu^{M\alpha-1}}{(1+Mu)^{(M+N)\alpha}}. \quad (3)$$

From this, we find that

$$H[R] \approx -N\langle u \log u \rangle \quad (4)$$

$$= \frac{N\alpha\Gamma(N\alpha-1)}{\Gamma(N\alpha)} (H_{N\alpha-2} + \log M - H_{M\alpha}) \quad (5)$$

$$= \log N + \log \alpha + \gamma + O(\frac{1}{N\alpha}) + \log M - H_{M\alpha} \quad (6)$$

which gives

$$I[X;R] = H[R] - \langle H[R|X=x] \rangle \quad (7)$$

$$= \log(N\alpha) + \gamma + O(\frac{1}{N\alpha}) + \log M - H_{M\alpha} - (\psi_0(N\alpha) - \psi_0(\alpha)) \quad (8)$$

where we have used $\psi_0(N\alpha) - \psi_0(\alpha)$ as the average entropy of the Dirichlet distribution with concentration parameter α and N elements. Using $\psi_0(N\alpha) = \log(N\alpha) + O(\frac{1}{N\alpha})$ we find that

$$I[X;R] \approx \gamma + \log M - H_{M\alpha} + \psi_0(\alpha) \quad (9)$$

$$= \log \alpha - \psi_0(\alpha) + O(\frac{1}{M\alpha}) \quad (10)$$

$$\approx \frac{1}{\alpha} + O(\frac{1}{M\alpha}) \quad (11)$$

which looks to be an increasing function of M. Note that this expression only holds for M large enough that $p(r)$ are i.i.d. with probability density function given approximately by $p(u)$ above.

The key points revealed by this analysis are that $I[X;R]$, while upper-bounded by $\log M$, actually saturates for random soft clusters of the data as $M,\ N \to \infty$ in a way that depends only on the randomness of the clustering α. Randomness, as one might expect, was solely detrimental. Numerical experiments above revealed that $I[X;R]$ did increase with N, but that there were diminishing returns to increases in the number of clusters.

B Plausibility argument for nonspecificity of large randomly-wired sensors

Recall that

$$I_{mem} = D_{KL}[p_{ss}(r,\sigma)||p_{ss}(r)p_{ss}(\sigma)]. \quad (12)$$

Rather than study I_{mem} directly, we instead study

$$I'_{mem} := D_{KL}[p_{ss}(r,\sigma)||\frac{1}{N}p_{ss}(\sigma)], \tag{13}$$

and it is straightforward to show that

$$I'_{mem} = I_{mem} + D_{KL}[p_{ss}(r)||\frac{1}{N}]. \tag{14}$$

Due to the nonnegativity of the Kullback-Leibler divergence, we have

$$I_{mem} \leq I'_{mem}, \tag{15}$$

which implies from an earlier Markov chain that

$$0 \leq I_{pred} \leq I_{mem} \leq I'_{mem}. \tag{16}$$

If we can argue, then, that I'_{mem} is $O(\frac{1}{N})$, we will have argued that $I_{pred},\ I_{mem}$ is $O(\frac{1}{N})$ as well.

We start with the case that $\vec{\alpha}$ is independent of state and input, i.e. $M^{(x)}_{:,\sigma}$ are i.i.d. draws from a Dirichlet distribution with concentration parameter $\vec{\alpha} = \alpha\vec{1}$. To start, we slightly rewrite the Chapman-Kolmogorov equations given in Methods as

$$p_{t+1}(r|\sigma) = \sum_{r',\sigma'} K_{(r,\sigma),(r',\sigma')} p_t(r'|\sigma') \tag{17}$$

with

$$K_{(r,\sigma),(r',\sigma')} := \sum_x M^{(x)}_{r,r'} T^{(x)}_{\sigma,\sigma'} \frac{p_{ss}(\sigma')}{p_{ss}(\sigma)}. \tag{18}$$

The desired steady-state distribution $p_{ss}(r|\sigma)$ is given by

$$p_{ss}(r|\sigma) = \lim_{t\to\infty} p_t(r|\sigma) \tag{19}$$

where $p_t(r|\sigma)$ can be initialized by anything that is a valid conditional probability distribution, i.e. $p_1(r|\sigma)$ is all nonnegative and $\sum_r p_1(r|\sigma) = 1$. We choose to study $p_t(r|\sigma)$ when $p_1(r|\sigma) = \frac{1}{N}$. One can show that, with this choice,

$$p_{t+1}(r|\sigma) = \frac{1}{N} \sum_{\sigma_1,r_1} \sum_{\sigma_2,r_2} \dots \sum_{\sigma_t,r_t} K_{(r_1,\sigma_1),(r_2,\sigma_2)} K_{(r_2,\sigma_2),(r_3,\sigma_3)} \dots K_{(r_{t-1},\sigma_{t-1}),(r_t,\sigma_t)}. \tag{20}$$

Though this is strictly not true, in the large N limit for t small enough (to be made precise in a second), we might argue that

$$p_{t+1}(r|\sigma) \approx \frac{1}{N} \prod_{i=1}^{t} u_i \tag{21}$$

with u_i being i.i.d. draws of a random variable U defined as a row sum of the random matrix with realizations K. (Since $\vec{\alpha} = \alpha\vec{1}$, each row sum has an identical distribution.) This approximation introduces a systematic error later on, in that the resulting $p_{t+1}(r|\sigma)$ are not valid conditional distributions. However, we can learn something about the size of the deviations of $p_{t+1}(r|\sigma)$ from $\frac{1}{N}$, which we then use to find the order of I'_{mem}. The random variable U is the ratio of two nearly independent Gamma

distributions, both with shape parameter $N\alpha$ and scale 1, which roughly leads to a probability density function of

$$p(u) = \int_0^\infty \int_0^\infty \delta(u - \frac{x}{y}) \frac{x^{N\alpha-1}e^{-x}}{\Gamma(N\alpha)} \frac{y^{N\alpha-1}e^{-y}}{\Gamma(N\alpha)} dxdy \quad (22)$$

$$= \int_0^\infty u^{N\alpha-1} \frac{y^{2N\alpha-1}e^{-(u+1)y}}{\Gamma(N\alpha)^2} dy \quad (23)$$

$$= \frac{\Gamma(2N\alpha)}{\Gamma(N\alpha)^2} \frac{u^{N\alpha-1}}{(1+u)^{2N\alpha}}. \quad (24)$$

The product of t of these factors, for t large, has a lognormal distribution since

$$\prod_{i=1}^t u_i = \exp(\sum_{i=1}^t \log u_i) \quad (25)$$

and by the Central Limit Theorem, $\sum_{i=1}^t \log u_i$ is normally distributed with mean and variance of the distribution $p(\log u)$ multiplied by t. As such, we calculate

$$\langle \log u \rangle = 0 \quad (26)$$

and

$$C_{N\alpha} := \langle (\log u)^2 \rangle = 2\psi_1(N\alpha) \quad (27)$$

to find that $p_{t+1}(r|\sigma)$ should be approximately lognormally distributed with parameters $\mu = 0,\ \sigma^2 = tC_{N\alpha}$. In the limit of large $N\alpha$, we have that $C_{N\alpha} \approx \frac{2}{N\alpha} + o(\frac{1}{N\alpha})$, so that $\sigma^2 \approx \frac{2t}{N\alpha}$. We therefore study the limit that $1 \gg t \gg N\alpha$, so that $p_t(r|\sigma)$ is nearly at its steady state distribution, but such that $\frac{t}{N\alpha} \ll 1$. If we are to take this lognormal distribution seriously, then the deviations in $p_t(r|\sigma)$ from $\frac{1}{N}$ are of size

$$\delta p(r|\sigma) = \frac{1}{N} O(e^{\mu+\frac{\sigma^2}{2}} - 1) + \frac{1}{N} O(\sqrt{(e^{\sigma^2}-1)e^{2\mu+\sigma^2}}) = O(\frac{1}{N\sqrt{N\alpha}}). \quad (28)$$

While this argument is quite loose, this scaling is confirmed numerically (not shown here). This implies that I'_{mem} is

$$I'_{mem} = D_{KL}[p_{ss}(\sigma)\left(\frac{1}{N} + \delta p(r|\sigma)\right) || p_{ss}(\sigma)\frac{1}{N}] \quad (29)$$

$$= \sum_{\sigma,r} p_{ss}(\sigma)\left(\frac{1}{N} + \delta p(r|\sigma)\right) \log\left(1 + N\delta p(r|\sigma)\right) \quad (30)$$

$$= \sum_{\sigma,r} p_{ss}(\sigma)\left(\frac{1}{N} + \delta p(r|\sigma)\right)\left(N\delta p(r|\sigma) - \frac{1}{2}(N\delta p(r|\sigma))^2 + O((N\delta p(r|\sigma))^3)\right) \quad (31)$$

$$= \frac{1}{2}\sum_{\sigma,r} N p_{ss}(\sigma)\delta p(r|\sigma)^2 + O(N^2 \delta p(r|\sigma)^3) \quad (32)$$

$$= O(\frac{1}{N\alpha}) \quad (33)$$

where we have used $\sum_r \delta p(r|\sigma) = 0$ for a correctly normalized conditional probability distribution and noted that the sum $\sum_r$ contributes a factor of N. This slope is indeed seen in Fig. 2 of the main text. The predictive information I_{pred} seems to decrease more quickly, as $O\left((\frac{1}{N\alpha})^2\right)$, and this remains unexplained.

We can extend this argument to the case in which $\vec{\alpha}$ is not $\alpha\vec{1}$ but instead is $\vec{\alpha}(x, r)$. That is, $M^{(x)}_{:,r}$ is drawn from a Dirichlet distribution with concentration parameters

$\vec{\alpha}(x,r)$. This case is more complicated in that it is not as immediately obvious that the steady-state distribution should be $\frac{1}{N}p(\sigma)$. We only provide a plausibility argument that this should be the steady-state distribution in the large N limit. As described in the main text, we need only confirm that the random variable defined by

$$U := \sum_{r'} \frac{Y_{r,r'}^{(x)}}{\sum_{r''} Y_{r'',r'}^{(x)}} \tag{34}$$

tends to 1 in the large N limit. The weak law of large numbers implies that $\sum_{r''} Y_{r'',r'}^{(x)}$ tends to $N\langle\alpha(x,r')\rangle$ with fluctuations of order $\sqrt{N}$, and so

$$U \approx \frac{1}{N}\sum_{r'} \frac{Y_{r,r'}^{(x)}}{\alpha(x,r')} + O(\frac{1}{\sqrt{N}}) \tag{35}$$

To show convergence of this in probability to 1, we use Chebyshev's inequality. We have

$$\mathbb{E}[U] = \frac{1}{N}\sum_{r'} \frac{\mathbb{E}[Y_{r,r'}^{(x)}]}{\alpha(x,r')} + O(\frac{1}{\sqrt{N}}) = 1 + O(\frac{1}{\sqrt{N}}) \tag{36}$$

and one can show that

$$\mathrm{Var}[U] \quad = \quad 1 + \frac{1}{N}\langle\frac{1}{\alpha}\rangle + O(\frac{1}{\sqrt{N}}), \tag{37}$$

which from Chebyshev's inequality then implies that

$$\lim_{N\to\infty} Pr(|U-1| > \epsilon) \leq \lim_{N\to\infty} \frac{\mathrm{Var}[U]}{\epsilon^2} = 0 \tag{38}$$

for any $\epsilon > 0$.

C Fluctuations in memory and prediction

Each randomly wired sensor leads to a slightly different value of memory I_{mem} and prediction I_{pred} for the same environment. Histograms not shown here suggest that the distribution of I_{pred} is roughly normal, with a slight asymmetry favoring higher values.

We find that the fluctuations in I_{pred} varies regularly with N and α. In Fig. 2, we plot the interquartile range of I_{pred} for that same environment as a function of α and N. That is, we calculate the value of I_{pred} that corresponds to the 75th percentile, and the value of I_{pred} that corresponds to the 25th percentile, and subtract the two. The interquartile range appears to decrease with both increasing N and increasing α. The decrease of the interquartile range with increasing N should be due to some sort of Central Limit Theorem-based argument, though proving as much can be quite difficult, e.g. see Ref. [2]. Similar statements hold for the interquartile range of I_{mem} (not shown).

References

1. Tishby N, Pereira FC, Bialek W. The information bottleneck method. In: The 37th annual Allerton Conference on Communication, Control, and Computing; 1999.

2. Marzen SE, DeDeo S. Weak universality in sensory tradeoffs. Phys Rev E. 2016;94(6):060101.

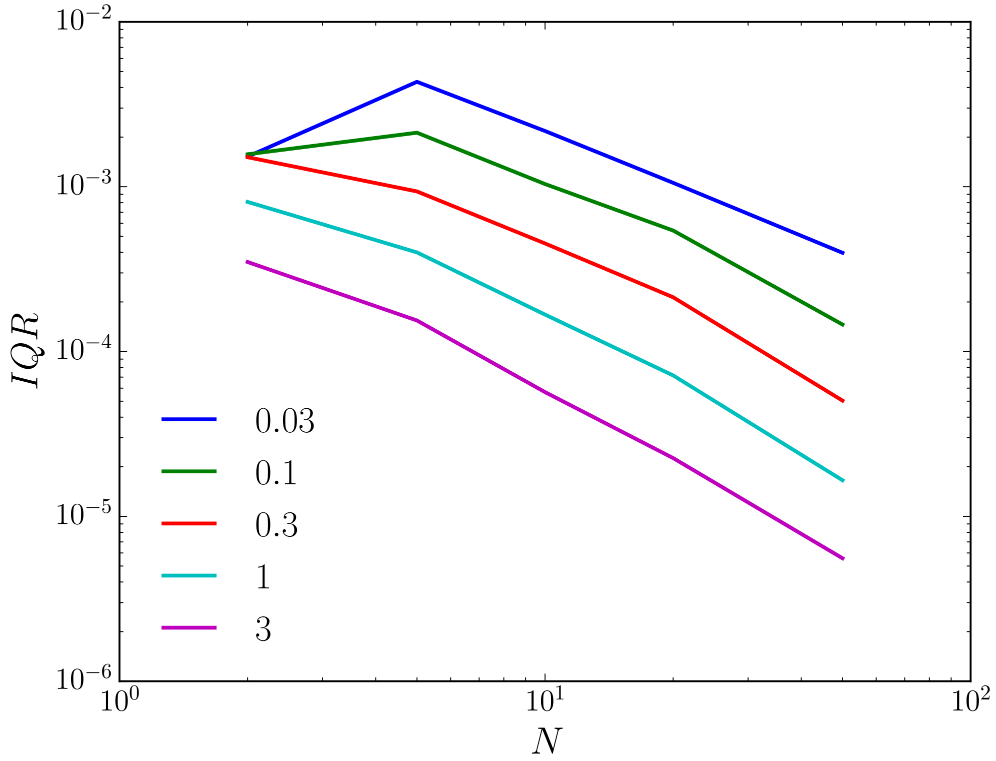

Fig 2. Variability in predictive information decreases with sensor size for fully nondeterministic sensors. On the x-axis is $|\mathcal{R}|$, or N, and on the y-axis is the interquartile range (IQR) of I_{pred}. The environment has $\rho_\mu = 0.147$ nats and $C_\mu = 2.36$ nats, but these results seemed to hold qualitatively regardless of particular environment.
