# Supplementary figures and images for: Infinitely large, randomly wired sensors cannot predict their input unless they are close to deterministic
Source: PLoS One. 2018 Aug 29;13(8):e0202333. doi: 10.1371/journal.pone.0202333 (PMC6114800; doi:10.1371/journal.pone.0202333)

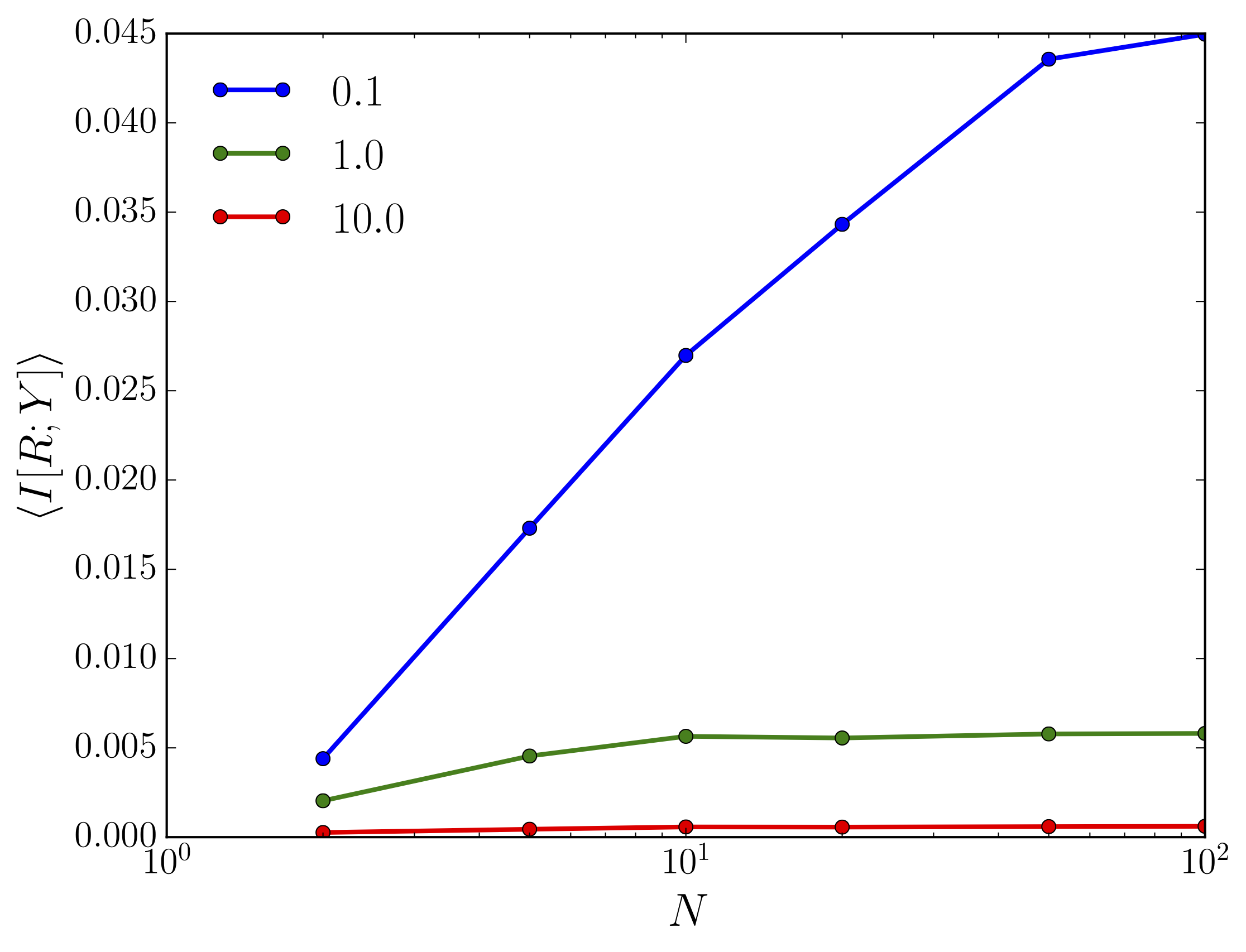

Supplement: S1 Fig — The average information obtained about the relevant variable Y. The various lines correspond to various values of α, as indicated in the legends, and the x-axis corresponds to variation in the number of clusters N. We chose M = 30. (TIFF) [file pone.0202333.s002.tiff]

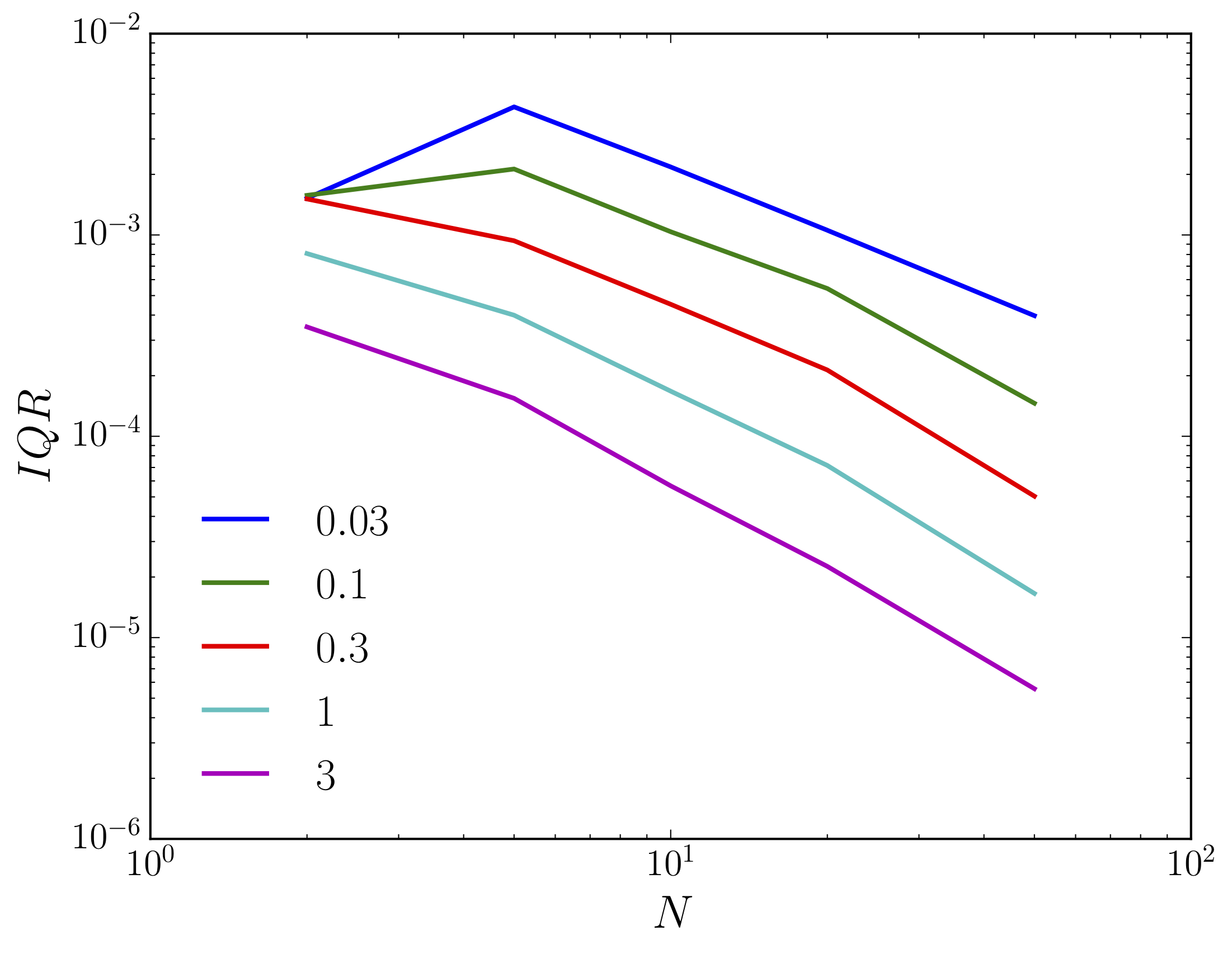

Supplement: S2 Fig — On the x-axis is |R|, or N, and on the y-axis is the interquartile range (IQR) of Ipred. The environment has ρμ = 0.147 nats and Cμ = 2.36 nats, but these results seemed to hold qualitatively regardless of particular environment. (TIFF) [file pone.0202333.s003.tiff]
